# Health and Social Care Inequalities During the First Wave of COVID-19 in Italy: Comment on "Experiences and Implications of the First Wave of the COVID-19 Emergency in Italy: A Social Science Perspective"

**DOI:** 10.34172/ijhpm.8717

**Published:** 2024-12-09

**Authors:** Fabrizio Pecoraro

**Affiliations:** Institute for Research on Population and Social Policies, National Research Council (IRPPS-CNR), Rome, Italy.

**Keywords:** COVID-19, Italy, Socio-Economic Impact, Population Health, Social Science, Territorial Divide

## Abstract

The impact of COVID-19 on the Italian population is well-known and has been deeply analysed under the clinical and epidemiological perspectives where the majority of the studies focused on the beginning of the first wave (March-May 2020). However, there is a need for analysing this complex phenomenon integrating the clinical side with the economic and social lens to better understand implications of a pandemic for populations. In their paper Masino and Enria focused the attention on four specific perspectives: health system reaction to the pandemic, inequalities in the work world, social care from the elderly point of view and the government communication challenges. In this commentary, I take these different perspectives trying to outline how they have been explored and analysed during these three years after their publication.

In their article: “Experiences and Implications of the First Wave of the COVID-19 Emergency in Italy: A Social Science Perspective”^1^ Masino and Enria examined the impact of the first wave of COVID-19 in Italy under the lens of socio-economic and political implications. Note that from a timeline perspective the paper was submitted in October 2021 and published in March 2023. This clearly frames the literature review published and referenced by authors at that time. On today’s date (July 2024), after more than four years from the first wave of the COVID-19, research studies have deeply analyzed the effect of the pandemic with an incredible volume of research under different perspectives. As outlined by Masino and Enria at the beginning of 2020 researchers solely focused the attention on transmission and epidemiological considerations with a huge number of clinical and laboratory studies published to investigate the coronavirus pandemic crises and to help policy-makers to understand how best to manage the current and future clinical and public health responses. Among this plethora of papers only a marginal number of studies took into account the social, political and economic dimensions, despite these perspectives allowed to provide a broader, more holistic perspective for effective pandemic management, particularly in the organization and analysis of outbreak responses in the stages following the immediate crisis.^2,3^ However, social scientists showed an increased interest in measuring social and community uneasiness in order to psychologically support the population, already at the beginning of 2020 and with a substantial increase during the subsequent phases of the virus spread.^4^ This discrepancy between the number of papers published in the clinical and social context can be associated with different causes among which the different speed in the availability, publication and production of quantitative and qualitative data. Since the onset of the COVID-19 pandemic in March 2020, national and international authorities began developing and updating datasets to supply data to researchers, journalists, and healthcare providers, as well as to inform the public.^5^ These datasets quickly became one of the primary information sources, with daily updates analyzed by scientists to study and predict the spread of the epidemic. Other quantitative aggregated data were already available for the research community and were adopted to analyse and appraise the response of national health services to COVID-19 on the basis of different aspects such as structural resources, policy and procedures adopted to mitigate the spread, etc. The availability of this data was one of the most crucial achievements during the early stages of the epidemic, aimed at supporting informed decision-making.^5^ Furthermore, this represented a significant improvement over previous efforts,^6-8^ both in the number of countries creating platforms to share COVID-19 data and in the timeliness of data publication, which greatly enhances its value for epidemiological analysis.^5^ Additionally, most countries are generating high-quality information daily, with a rise in innovative datasets that have provided unique insights since the pandemic began.^5^ On the contrary, different challenges may rise in the collection of qualitative data. As reported by Tremblay et al,^9^ conducting qualitative investigations during a pandemic requires the union between two interrelated aspects: adaptation, while maintaining a high quality of research. They identified two main challenges: time constraints and physical distancing that may both threaten qualitative research standards. At the beginning of the pandemic researchers working on almost all disciplines were put under pressure to produce time-sensitive responses to the pandemic qualitative research in a short window of time with faster design, recruitment of participants, and data collection and analysis. The second challenge concerns physical (or social) distancing due to the lockdown and restrictive measures adopted by national and local government to halt the spread of the pandemic. This physical distancing measure has encouraged researchers to explore alternative, innovative data-gathering methods that leverage technology for virtual interactions, both synchronous and asynchronous, such as online focus groups, interviews, digital text communications, and written or video diaries. Despite their long-standing use and recent surge in popularity, virtual interaction methods still raise concerns regarding methodological rigor.^10^ Time and physical distancing are only two of the main challenges to be faced during a pandemic period to collect qualitative data and that may have had an impact with the low number of studies published under the social science perspective.

 Despite these issues, under the methodological point of view, Masino and Enria collected a set of semi-structured interviews and written testimonies already at the beginning of the COVID-19 spread (ie, starting in March 2020), engaging 13 interviews in the southern regions and 16 testimonies coming from the northern regions. The sample size (13 interviews and 16 testimonies) might be considered too small to draw broad conclusions about potential regional differences, especially since it does not represent all Italian regions, having excluded central Italy. Additionally, whether differing methodologies between the south and north could introduce a bias and serve as another limitation of the study is not addressed. However, despite these limitations, results reported by Masino and Enria underline the different experiences of citizens coming from the northern and southern regions. In Italy the north-south divide in the economic^11^ and provision of services^12^ is well-known and became more impactful during the COVID-19.^13^

## Impact of COVID-19: Four Different Perspectives

 Interviews and testimonies along which authors comments and studies published in the literature were adopted to analyse the response of the Italian and the regional health systems under four main perspectives: health system, economy, social care, and social response.

 Firstly, authors analysed the different responses of the regional health systems to COVID-19 comparing the north and the south part of the country in relation to the availability of hospital and primary care resources. Despite the south of the country lacks in the number of beds per population compared to the northern regions, the different onset of the pandemic as well as the home-hospital mixed approach in managing COVID-19 patients^14^ have avoided the saturation of the hospital beds (both general and in the intensive care unit) in the majority of the central-southern regions even if the number of inpatients increased over time and the availability of beds were significantly lower than in the northern part of the country.^15^ For instance, Figure shows the occupancy rate of the intensive care unit beds to 29th of March 2020, highlighting that all regions that saturated (Lombardia, Piemonte, Val d’Aosta, Trento, and Bolzano) or almost saturated (Marche, Emilia Romagna, Toscana, and Liguria) the capacity of intensive care unit beds was located in the northern part of Italy.

**Figure:**
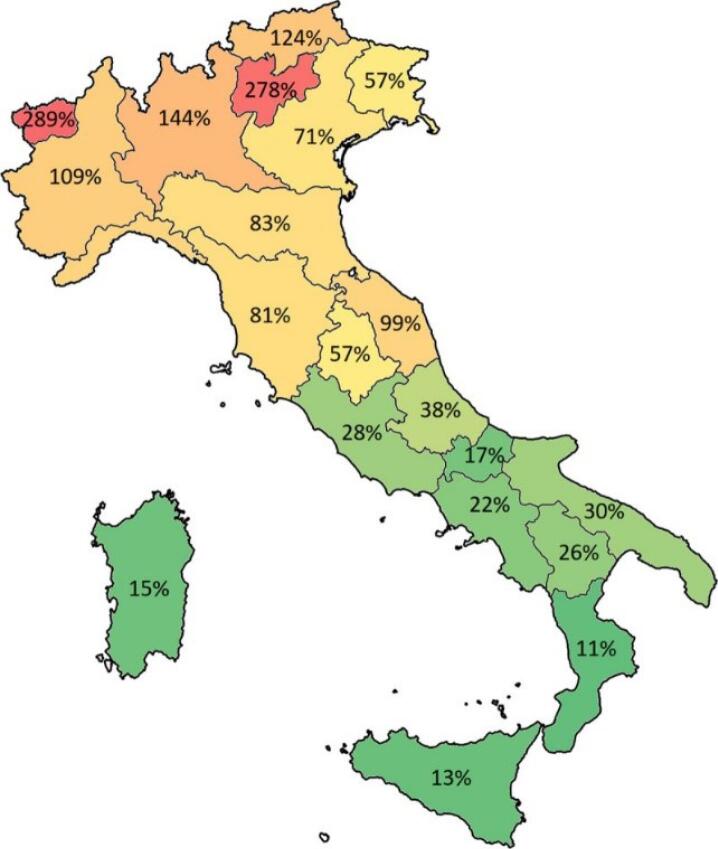
Percent of Number of Intensive Care Unit Beds Occupied by COVID-19 Patients by Region, Based on Routinely Available Beds. Source: Pecoraro et al.^15^

 Various approaches to treating coronavirus patients were observed in northern Italy. In Lombardy, the care model relied heavily on a network of public and private hospitals, resulting in significant hospitalizations. In contrast, Veneto adopted a comprehensive community-based strategy, leveraging its strong public health network and local service integration.^14^ This divergence may have affected virus spread within hospitals and influenced mortality rates among healthcare workers, patients with other conditions, and healthy individuals.^14^ The availability of personal protective equipment also played a crucial role in the virus’s transmission, with shortages reported by general practitioners. These findings highlight that, despite ongoing debates about the best strategies to address the pandemic, community management models are among the most effective responses. Successful implementation requires not only robust formal health services—such as primary care physicians and community nurses—but also the involvement of informal home healthcare providers, like family members, who are vital in meeting patients’ specific needs and serving as frontline leaders during the crisis.

 The second theme discussed by Masino and Enria entailed the striking inequalities of the first wave of the coronavirus crisis focusing the attention on the most affected business sectors and working categories, such as tourism, entertainment, factory (ie, production), cultural, recreation. In 2020, as stated by the Italian National Institute of Statistics, Italy lost around 724 000 jobs. The lockdown established by the national government in March 9, 2020 and continued until June 15, 2020 (with the exception of those deemed “essential” for the functioning of the country’s economic system) and the consequent drop of Italy’s gross domestic product (ie, about 9% in 2020^1^) critically impacted on loss the of workers with the majority of them among temporary and self-employed workers, both in terms of turnover and employment due to the implemented restrictions and the inability of workers to work from home.^16^ These inequalities were particularly exacerbated in the most disadvantaged society groups, such as migrants and the poorest social classes, women, young people and foreigners. Despite authors did not discuss such inequalities from a territorial perspective, the majority of the testimonies were from the Campania region (ie, located in the south of Italy) and employed in dependent jobs (eg, hairdresser informally employed, pastry chef). These aspects represent an important limitation of the originality of the study even if the prevalence of informal workers in the Campania region suggests significant differences across regions and a north-south divide on this issue. Moreover, they mainly reported the impact of COVID-19 on the employment procedures raising issues in the definition of a formal contract or the ability to get a job due to postponed surgery procedure. Employing new workers as well as renew fixed-term contracts also worried employees as underlined by a local non-governmental organization coordinator in the Campania region, in particular considering work as carers or cleaners who worked informally before the pandemic and during the lockdown must justify going out without a contract. Although the authors did not include a gender perspective in their analysis, various studies have shown that the pandemic intensified pre-existing employment inequalities, particularly when intersecting with other socioeconomic disadvantages. Single mothers and women with lower educational levels were more impacted than their male counterparts, whereas single men without children and foreign men faced greater challenges than women with similar backgrounds in the immediate aftermath of the pandemic.^17^

 The third perspective analysed by Masino and Enria focused on the trustiness of citizens towards governmental and non-governmental institutions, with a particular attention on communication challenges: contradictions in the ordinances, lack of information, disagreement among experts, and authorities, etc. Building trust in the national response during the first wave of the pandemic was particularly challenged not only due to this conflicting and confused communication, but also by contradictory protocols, for instance, in testing procedures to be carried out at regional and local level. We have already discussed about the differences between regions in testing or not asymptomatic patients. Despite these results and that two-thirds of the population tended not to trust the government,^1^ Italians were inclined to respect the lockdown and restrictive measures imposed by the central or the local governments.^18^ Indeed, one of the most worrying aspects was the limitation of social interactions together with mental health issues, with some differences between age classes: as reported in Bouckaert et al^19^ it took longer to convince young people (ie, less than 30 years) to respect the lockdown measures. Additionally, older adults (ie, 65-85 years) exhibited fewer negative emotions, expressed greater confidence in the COVID-19–related information they received, were more supportive of the restrictive measures, perceived the emergency as less underestimated, and maintained a more positive outlook on the situation compared to other age groups.^20^

 The four and last perspective posed the attention on the social care component with particular attention on frail elderly people. Difficulties from a psychological point of view were outlined by a gym instructor who reported the permanent state of anxiety of his parents who lived alone as they are separated. This crucial aspect of the impact of COVID-19 on our lives was superficially treated by authors underlining only the paternalistic/maternalistic role of elderlies in the society in particular in supporting the whole family as primary providers of childcare. Moreover, other age groups such as youth and adolescents had to be taken into consideration considering their frailness due to the lack of social interactions and sport practice. Additional topics related to social care have been addressed by Masino and Enria in their paper, such as institutional trust, communication challenges, social cohesion and organized crime. However, these themes have been discussed only briefly by authors mainly focusing the attention on the sense of community belonging, for instance, by respecting governmental rules (eg, lockdown) despite trust in institutions is historically low in Italy.

## Conclusion

 During the first months of the pandemic, it was clear that the clinical and epidemiological perspectives were not sufficient to analyse this unexpected phenomenon. Analyzing public health crises like the COVID-19 pandemic through socio-economic and political lenses is essential for fully understanding the experiences and implications involved. This approach helps in developing effective response measures that are both locally relevant and widely accepted. Different reasons have tipped the balance towards the clinical domain among which the availability and the quality of quantitative data, compared to qualitative ones. However, to date and also in the first months of 2020, different studies have posed the attention on the social side of life through the establishment of multi-disciplinary task forces to render policy-making and social support interventions as well as communication strategies more effective. In their work, Masino and Enria collected a set of interviews and narratives to better understand the critical issues that emerged in real life during the first phase of the pandemic. They analysed such experiences and implications considering four main perspectives trying to capture differences between the north and the south of the country. Despite the limited sample of interviewees involved and the rapid methodology adopted to collect and analyse the narratives, authors provided an interesting view on how the COVID-19 has impacted our lives under the socio-economic perspective. Particularly interesting is the focus on the impact of the virus on the working world highlighting, through testimonies from the south of the country, the inequalities not only between the socio-economic and demographic status, but also considering the business sectors that were most affected by lockdown policies. Social care perspective, unfortunately, were discussed only marginally and focusing on the elderly, without considering the impact of lockdown and other restrictions on children and adolescents who did not go to school, played sports and met friends with an undoubted significant impact on the lifestyle and psychological well-being of Italian adolescents.^21^

## Ethical issues

 Not applicable.

## Conflicts of interest

 Author declares that he has no conflicts of interest.
